# Supplementary material for: Analysis of PPARγ Signaling Activity in Psoriasis
Source: Int J Mol Sci. 2021 Aug 10;22(16):8603. doi: 10.3390/ijms22168603 (PMC8395241; doi:10.3390/ijms22168603)
Supplement: Supplementary file 1 [file ijms-22-08603-s001.zip › Supplemental materials_Analysis of PPARg signaling activity in psoriasis/Pathway models/Models images and html files/Model of PPARG related pathways in psoriasis (short version)/9773.html]

RASGRF1


# Protein RASGRF1

|  |  |
| --- | --- |
| URN | urn:agi-llid:5923 |
| Total Entities | 0 |
| Connectivity | 404 |
| Name | RASGRF1 |
| Description | Ras protein-specific guanine nucleotide-releasing factor 1 |
| Notes | The protein encoded by this gene is a guanine nucleotide exchange factor (GEF) similar to the Saccharomyces cerevisiae CDC25 gene product. Functional analysis has demonstrated that this protein stimulates the dissociation of GDP from RAS protein. The studies of the similar gene in mouse suggested that the Ras-GEF activity of this protein in brain can be activated by Ca2+ influx, muscarinic receptors, and G protein beta-gamma subunit. Mouse studies also indicated that the Ras-GEF signaling pathway mediated by this protein may be important for long-term memory. Alternatively spliced transcript variants encoding distinct isoforms have been reported. [provided by RefSeq, Mar 2009] |
| Primary Cell Localization | Cytoplasm |

---

|  |  |
| --- | --- |
| Pathway | Skin Aging |
|  | High Fat Diet Activates cAMP Related Exocytosis in Synapse and Endocrine Cell |
|  | mTOR Signaling Activation by Taste Sensor Receptors (Rodent Model) |
|  | Dopamine Receptors Signaling |
|  | Serotonin Receptors Signaling |
|  | Catecholamines Secretion from Adrenal Gland |
|  | CRH Secretion Regulation |
|  | Prolactin Production |
|  | Thrombopoietin Receptors Signaling in Platelet Maturation |
|  | Mineralcorticoids Secretion Regulation |
|  | Plasmin Effects in Inflammation |
|  | Proplatelet Maturation |
|  | Platelet Activation via GPCR Signaling |
|  | Leukotriene Effect on Vascular Endothelial Cell Response |
|  | Leukotriene Effect on Airway Smooth Muscle Response |
|  | Mast-Cells De Novo Synthesized Mediators via IgE Independent Signaling |
|  | CD16/CD14 Proinflammatory Monocyte Activation |
|  | anti-Inflammatory Function of Macrophage M2 Lineage |
|  | CR3 Mediated Phagocytosis in Neutrophils and Macrophages |
|  | Vascular Endothelial Cell Activation by Blood Coagulation Factors |
|  | Vascular Endothelial Cell Permeability Activation |
|  | Nociception Expression Targets Signaling |
|  | Vascular Motility |
|  | NPY1R -> CRH/POMC Production |
|  | NTSR1 Expression Targets |
|  | EDNRA/B -> Vascular Motility |
|  | ADRA1 -> Vasoconstriction |
|  | ADRA1A -> IL6 Production |
|  | ADRA2C/ADRB2 -> Vasoconstriction |
|  | ADRA2C/ADRB2 -> Synaptic Endocytosis |
|  | ADRA1 -> Prostaglandin Generation |
|  | BDKRB1/2 -> Prostaglandin Generation |
|  | CNR1/2 -> IL1B/2/4/6/10 Production |
|  | DRD2 Expression Targets |
|  | GALR1/2/3 -> POMC/NPY Production |
|  | HRH2/4 -> IL6/10 Production |
|  | CHRM1 -> IL2 Production |
|  | CHRM1/2/3 -> Vascular Motility |
|  | OPRK -> Pain Perception |
|  | PTGIR -> IL6 Production |
|  | HTR1 -> IL6 Production |
|  | HTR5 -> TNF Production |
|  | HTR1 -> Membrane Transport |
|  | HTR1 -> Vascular Motility |
|  | P2RY11/13/14 -> IL8/10 Production |
|  | Luteinizing Hormone in Oocyte Meiotic Resumption |
|  | G0/G1 Phase Transition |
|  | Cardiomyocyte Hypertrophy |
|  | Hypertrophic Cardiomyopathy |
|  | Endothelin Signaling in Arterial Hypertension |
|  | Aldosterone Synthesis Acute Regulation |
|  | Smooth Muscle Cell Dysfunction in Arterial Hypertension |
|  | TRPM4/6/7/8 Signaling in Arterial Hypertension (Hypothesis) |
|  | Smooth Muscle Cell Dysfunction in Pulmonary Hypertension |
|  | Myocardial Remodeling |
|  | Contraction Due Vasospasm |
|  | B-Cell Activation in Systemic Lupus Erythematosus |
|  | Vitiligo |
|  | Apoptosis of Melanocyte in Vitiligo |
|  | Proliferative Diabetic Retinopathy |
|  | G-Proteins Signaling in Insulin Secretion in beta-Cell |
|  | Olanzapine Effect on Insulin Secretion in Diabetes Mellitus |
|  | Ghrelin Influence on Insulin Secretion (Hypothesis) |
|  | RET1 Mutation in Hyperparathyroidism |
|  | Hypothyrodism Associated Hyperprolactinemia |
|  | DRD2 Pharmacological Inhibition in Hyperprolactinemia |
|  | Ethanol Induced Efects in Hyperprolactinemia |
|  | GNRH Signaling Impairment in Hypogonadotrophic Hypogonadism |
|  | Prolactinoma Overiew |
|  | EDN, NRG, NRTN, and GDNF/RET Signaling in Hirschsprung Disease |
|  | Hodgkin Lymphoma |
|  | T-Cell Acute Lymphoblastic Leukemia |
|  | Airway Smooth Muscle Cell High Rate Proliferation |
|  | IgE Induces Airway Smooth Muscle Cell Proliferation |
|  | Airway Smooth Muscle Cell Contraction |
|  | Mast-Cell Related Chemokines Signaling in Asthma |
|  | Steroidogenesis Impairement in Polycystic Ovary Syndrome |
|  | Ovulation Block |
|  | FSH Action in Polycystic Ovary Syndrome |
|  | Dentin Formation Disruption |
|  | Proteins Involved in Epilepsy |
|  | BDNF Upregulation Triggered by Seizures |
|  | Apoptosis and Immediate Early Gene Activation in Epileptiform Disorders |
|  | Vascular Smooth Muscle Cell Vasodilation in Brain |
|  | NO Effect on Vasodilation and Vasoconstriction |
|  | Angiogenesis in Endometriosis |
|  | Coagulation Factors and PAR1/2 Receptors (F2R/F2RL1) in Endometriosis |
|  | EGFR and PTAFR Signaling in Ovarian Cancer |
|  | HPV E5 Regulates EDNRA and EGFR Signaling Pathway in Keratinocytes |
|  | VEGFA in Neovascular Glaucoma |
|  | PGF2-alpha Effect on Treatment |
|  | Cytokines Trigger Otitis Media |
|  | Muscarinic Receptor-Mediated Airway Smooth Muscle Contraction |
|  | Polycystins Mutation Effects in Cystic Kidney Disease |
|  | Plasmin Role in Glomerulonephritis |
|  | Sertoli Cells Dysfunction Causes Male Infertility |
|  | CD38/CD3 -> JUN/FOS/NF-kB Signaling in T-cell Proliferation |
|  | CD38/CD19 -> JUN/FOS/NF-kB Signaling in B-cell Proliferation |
|  | CD38 -> cADPR/Calcium Signaling |
|  | CD157 -> cADPR/Calcium Signaling |
|  | ANGPT1 -> CREB/CREBBP Expression Targets |
|  | ANGPT2 -> AP-1/CREBBP/MYC Expression Targets |
|  | AREG -> CREB/CREBBP Expression Targets |
|  | AREG -> NCOR2 Expression Targets |
|  | AREG -> SMAD1 Expression Target |
|  | AREG -> HIF1A Expression Targets |
|  | AREG -> AP-1 Expression Targets |
|  | Noradrenaline/Gs Expression Targets |
|  | Noreadrenaline/Gq Expression Targets |
|  | Epinephrine/Gs Expression Targets |
|  | Acetylcholine Expression Targets |
|  | Serotonin/Gq Expression Targets |
|  | Epinephrine/Gq Expression Targets |
|  | Dopamine/Gi Expression Targets |
|  | Aryl Hydrocarbon Receptor Genomic and non-Genomic Signaling |
|  | B-Cell Receptor -> AP-1 Signaling |
|  | BTC -> AP-1/ATF/CREB Expression Targets |
|  | BTC -> EP300/ETS/ETV/SP1 Expression Targets |
|  | SELE -> ELK-SRF Signaling |
|  | CD19 Expression Targets |
|  | CD81 -> AP-1 Expression Targets |
|  | CD247 Expression Targets |
|  | gamma Globulins Expression Targets |
|  | FCGR3A Expression Targets |
|  | CD19/CR2 Expression Targets |
|  | CD72 -> CREB/CREBBP Expression Targets |
|  | CD8 -> ATF/CREB/CREBBP Expression Targets |
|  | CD80 -> NF-kB Expression Targets |
|  | CD86 -> NF-kB Expression Targets |
|  | CXCL6 Expression Targets |
|  | CXCL1 Expression Targets |
|  | CXCL2 Expression Targets |
|  | CXCL3 Expression Targets |
|  | CXCL5 Expression Targets |
|  | Complement Component GPCRs Signaling |
|  | Clozapine Induced Granulocytopenia |
|  | EGF -> HIF1A Expression Targets |
|  | EGF -> AP-1/ATF Expression Targets |
|  | EGF -> CREB/CREBBP/ELK/SRF/MYC Expression Targets |
|  | EGF -> TP53 Expression Targets |
|  | EGF -> NCOR2 Expression Target |
|  | EGFR -> NCOR2 Signaling |
|  | EGFR/ERBB2 -> TP53 Signaling |
|  | ERBB2/3 -> EP300/ETS/ETV/SP1 Signaling |
|  | EGFR -> SMAD1 Signaling |
|  | EGFR/ERBB2 -> HIF1A Signaling |
|  | EGFR -> AP-1/ATF2 Signaling |
|  | EGFR -> AP-1/CREB/ELK/SRF/MYC Signaling |
|  | EREG -> CREB Expression Target |
|  | EREG -> EP300/SP1 Expression Targets |
|  | EREG -> HIF1A Expression Target |
|  | EREG -> AP-1/ATF Expression Targets |
|  | Erythropoietin -> AP-1/MYC/CREB Expression Targets |
|  | Erythropoietin -> ELK/SRF Expression Targets |
|  | PECAM1 Expression Targets |
|  | F2 -> AP-1/CREB/ELK/SRF/SP1 Expression Targets |
|  | PLG -> AP-1/CREB/ELK/SRF/SP1 Expression Targets |
|  | FGF9 -> AP-1/CREB/MYC Expression Targets |
|  | FGF10 -> AP-1/CREB/CREBBP/MYC Expression Targets |
|  | FGF1 -> AP-1/CREB/ELK/SRF/MYC Expression Targets |
|  | FGF1 -> NCOR2 Expression Target |
|  | FGF7 -> RUNX Expression Targets |
|  | FGF8 -> AP-1/CREB/MYC Expression Targets |
|  | FGF1 -> RUNX Expression Targets |
|  | FGF18 -> AP-1/CREB Expression Targets |
|  | FGF23 -> NCOR2 Expression Targets |
|  | FGF8 -> RUNX Expression Targets |
|  | FGF9 -> RUNX Expression Targets |
|  | FGF2 -> NCOR2 Expression Targets |
|  | FGF4 -> AP-1/MYC Expression Targets |
|  | FGF7 -> AP-1/CREB/CREBBP/MYC Expression Targets |
|  | FGF2 -> RUNX Expression Targets |
|  | FGF2 -> AP-1/CREB/CREBBP/ELK/SRF/MYC Expression Targets |
|  | FGFR -> RUNX2 Signaling |
|  | FGFR -> AP-1/CREB/CREBBP/ELK/SRF/MYC Signaling |
|  | ADCYAP1 Expression Targets |
|  | FSHR Expression Targets |
|  | Glutamate/Gq Expression Targets |
|  | VIP Expression Targets |
|  | HBEGF -> AP-1/ATF Expression Targets |
|  | HBEGF -> TP53 Expression Targets |
|  | HBEGF -> EP300/ETS/ETV/SP1 Expression Targets |
|  | HBEGF -> HIF1A Expression Targets |
|  | HBEGF -> CREB/MYC Expression Targets |
|  | HGF -> AP-1/CREB/ELK/SRF/MYC Expression Targets |
|  | HGFR -> AP-1/CREB/MYC Signaling |
|  | TAM Receptor Signaling Activation in Cancer |
|  | G0/G1 Cell Cycle Phase Transition Activation in Cancer |
|  | VEGF Independent Angiogenesis in Cancer |
|  | CFL1 in Cancer Cell Motility |
|  | CD19 -> JUN/ELK Signaling |
|  | FcIgER -> ELK-SRF Signaling |
|  | ICAM1 -> AP-1/CREB/ELK/SRF Signaling |
|  | FibronectinR -> AP-1/ELK/SRF/SREBF Signaling |
|  | CTGF -> AP-1/CREB/MYC Expression Targets |
|  | CTGF -> NCOR2 Expression Target |
|  | ICAM1 Expression Targets |
|  | IL16 -> AP-1 Expression Targets |
|  | UrokinaseR -> ELK/SRF Signaling |
|  | PTPRC -> BCL6 Expression Targets |
|  | Prostaglandin F Expression Targets |
|  | Thromboxane A2 Expression Targets |
|  | LPA Expression Targets |
|  | PAF/Gq -> AP-1/ATF1/CREB/ERK/SRF Expression Targets |
|  | Dronabinol/Anandamide Expression Targets |
|  | Morphine Expression Targets |
|  | PAF/Gq -> NF-kB Expression Targets |
|  | S1P Expression Targets |
|  | PGE1 Expression Targets |
|  | NRG1 -> EP300/ETS/ETV/SP1 Expression Targets |
|  | NRG1 -> CREB/CREBBP/ELK/SRF/MYC Expression Targets |
|  | NRG1 -> TP53 Expression Target |
|  | NRG1 -> AP-1/ATF Expression Targets |
|  | NRG1 -> HIF1A Expression Target |
|  | NGF -> SMAD3/NF-kB Expression Targets |
|  | NGF -> AP-1/TP53/MYC Expression Targets |
|  | NGF -> FOXO/MYCN/ELK-SRF Expression Targets |
|  | BDNF Expression Targets |
|  | NTF3 Expression Targets |
|  | NTF4 Expression Targets |
|  | NGF -> CREB/CEBPB/MEF2A Expression Targets |
|  | Adenosine Expression Targets |
|  | PDGFB -> AP-1/CREB/MYC Expression Target |
|  | PDGFC -> CREB Expression Target |
|  | PDGFD -> AP-1 Expression Targets |
|  | PDGF -> AP-1/CREB/CREBBP/MYC Expression Targets |
|  | KIT -> MITF Signaling |
|  | PDGFR -> AP-1/MYC Signaling |
|  | AVP/Gs -> CREB/ELK/SRF/AP-1/EGR Expression Targets |
|  | GNRH1 Expression Targets |
|  | NTS Expression Targets |
|  | GAST Expression Targets in GAST Expression Targets |
|  | AGT -> TP53 Expression Targets |
|  | GNRH2 Expression Targets |
|  | OXT Expression Targets |
|  | POMC Expression Targets |
|  | CCL11 Expression Targets |
|  | EDN1 Expression Targets |
|  | AGT -> CREB Expression Targets |
|  | NPY Expression Targets |
|  | IFNA1/Gq Expression Targets |
|  | AGT -> ELK/SRF Expression Targets |
|  | TAC1 Expression Targets |
|  | EDN3 Expression Targets |
|  | AVP/Gq -> CREB/ELK/SRF/AP-1/EGR Expression Targets |
|  | PDCD1 -> AP-1 Expression Targets |
|  | PTPRC -> BCL6 Signaling |
|  | AngiopoietinR -> AP-1 Signaling |
|  | GDNF -> HSF1 Signaling |
|  | NTRK -> AP-1/CREB/ELK/SRF/MYC/SMAD3/TP53 Signaling |
|  | GAS6 -> AP-1/CREB Expression Targets |
|  | FIGF -> AP-1 Expression Target |
|  | FIGF -> NCOR2 Expression Target |
|  | KITLG -> AP-1/CREB/CREBBP/MYC Expression Targets |
|  | GDNF -> HSF1 Expression Targets |
|  | CSF1 -> AP-1/CREB/CREBBP/MYC Expression Targets |
|  | PGF -> AP-1/CREB/CREBBP/MYC Expression Targets |
|  | FLT3LG -> AP-1/CREB/CREBBP Expression Targets |
|  | Lysophosphatidic Acid/LPARs Signaling |
|  | Ras-GRF Regulation Signaling |
|  | MERTK Signaling |
|  | AMPK Signaling |
|  | AXL Receptor Tyrosine Kinase Signaling |
|  | EGFR Signaling |
|  | HRAS Signaling |
|  | TCR -> AP-1 Expression Targets |
|  | TGFA -> TP53 Expression Targets |
|  | TGFA -> HIF1A Expression Targets |
|  | TGFA/AP-1/ATF Expression Targets |
|  | TGFA -> CREB/CREBBP/ELK-SRF/MYC Expression Targets |
|  | Thrombopoietin -> AP-1/CREB/CREBBP/MYC Expression Targets |
|  | ROS in Triggering Vascular Inflammation |
|  | ROS and RNS in Vasoconstriction and Vasodilation Regulation |
|  | ROS in Angiotensin Mediated Cardiovascular Remodeling and Hypertrophy |
|  | PLAU -> ELK-SRF/AP-1 Expression Targets |
|  | VEGFA -> ATF/CREB/ELK-SRF Expression Targets |
|  | VEGFA -> NFATC Expression Targets |
|  | VEGFC -> ATF Expression Target |
|  | VEGFA -> AP-1/CREBBP/MYC Expression Targets |
|  | VEGFA -> NCOR2 Expression Target |
|  | VEGFR -> NFATC Signaling |
|  | VEGFR -> AP-1/CREB/MYC Signaling |
|  | VEGFR -> ATF/CREB/ELK-SRF Signaling |
|  | pathway 3 |
|  | Model of PPARG signaling in psoriasis |
|  | Model of PPARG related pathways in psoriasis (short version) |
|  | 3\_Enhanced angiogenesis in endometriosis |
|  | 1\_Disruption of luteinizing hormone and follicle-stimulating hormone secretion in PCOS |
|  | 2\_Cardiomyocyte hypertrophy |
|  | 3\_Dysfunction of smooth muscle cells in hypertension |
|  | 1\_Rise in aldosterone synthesis in adrenal zona glomerulosa cells |
|  | 2\_The intense proliferation and contractile activity of smooth muscle cells in pulmonary hypertension |
|  | 3\_1\_Mucus accumulation in asthma |
|  | 4\_Mucin hyperproduction in goblet and mucous cells in COPD |
|  | 1\_2\_Insulin synthesis and secretion: insulin secretion |
|  | 1\_1\_Insulin synthesis and secretion: insulin processing and storage |
|  | 3\_2\_Beta-cell proliferation and compensation: neogenesis/dedifferentiation |

---

|  |  |
| --- | --- |
| Source | curated |
|  | GO component |

---

|  |  |
| --- | --- |
| MedScan ID | 5923 |
|  | 192213 |

---

|  |  |
| --- | --- |
| LocusLink ID | 5923 |
|  | 19417 |
|  | 192213 |
|  | 26449 |
|  | 9983 |
|  | 315877 |
|  | 785619 |
|  | 568885 |

---

|  |  |
| --- | --- |
| Alias | LOC315877 |
|  | LOC145899 |
|  | PP13187 |
|  | Ras-specific nucleotide exchange factor CDC25 |
|  | Rasgrf I |
|  | GRFbeta |
|  | GRF55 |
|  | AI844718 |
|  | guanine nucleotide-releasing factor, 55 kD |
|  | RASGRF1 |
|  | Ras guanine release factor beta |
|  | Ras-specific guanine nucleotide-releasing factor, CDC25 homolog |
|  | Ras protein-specific guanine nucleotide-releasing factor I |
|  | guanine nucleotide exchange factor (S. cerevisiae CDC25-related, son of sevenless-related) |
|  | guanine nucleotide releasing factor 1 |
|  | CDC25Mm |
|  | P140 Ras-GRF |
|  | Ras guanine nucleotide-releasing factor 1 |
|  | Ras-GRF1 |
|  | Ras guanine nucleotide-releasing factor I |
|  | H-GRF55 |
|  | Ras protein-specific guanine nucleotide-releasing factor 1 |
|  | GNRP |
|  | GRF1 |
|  | CDC25 |
|  | CDC25L |
|  | guanine nucleotide exchange factor |
|  | guanine nucleotide-releasing factor 1 |
|  | guanine nucleotide-releasing protein |
|  | ras-specific guanine nucleotide-releasing factor 1 |
|  | p190 |
|  | P190-A |
|  | p190RhoGEF |
|  | Ras-specific guanine nucleotide-releasing factor, homolog |
|  | cdc25 homolog |
|  | Guanine nucleotide releasing protein (GNRP) (P140 Ras-GRF) |
|  | GRF beta |
|  | p190Rho-GAP |
|  | p190Rho-GEF |
|  | guanine nucleotide exchange factor(S. cerevisiae CDC25-related, son of seve |
|  | guanine nucleotide exchange factor(S. cerevisiae CDC25-related, son of sevenless-related) |
|  | RAS guanyl releasing protein 3 |
|  | Rasgrp3 |
|  | Ras-specific nucleotide exchange factor CDC25 |
|  | RAS guanyl releasing protein III |
|  | Guanine nucleotide releasing protein |

---

|  |  |
| --- | --- |
| GO ID | 0005088 |
|  | 0005089 |
|  | 0035254 |
|  | 0005085 |
|  | 0000165 |
|  | 0090630 |
|  | 0008283 |
|  | 0007616 |
|  | 0031175 |
|  | 0043547 |
|  | 0046579 |
|  | 2000310 |
|  | 0035020 |
|  | 0046578 |
|  | 0035023 |
|  | 0048168 |
|  | 0048167 |
|  | 0034976 |
|  | 0007165 |
|  | 0007264 |
|  | 0005829 |
|  | 0030426 |
|  | 0043005 |
|  | 0005886 |
|  | 0017016 |
|  | 0008022 |
|  | 0019901 |
|  | 0030971 |
|  | 0035556 |
|  | 0048146 |
|  | 0097440 |
|  | 0016327 |
|  | 0016323 |
|  | 0005737 |
|  | 0043025 |
|  | 0005096 |
|  | 0071236 |
|  | 0032869 |
|  | 0001975 |
|  | 0005622 |
|  | 0032863 |
|  | 0032320 |
|  | 0007268 |
|  | 0051056 |
|  | 0005099 |
|  | 0005543 |
|  | 0006915 |
|  | 0048011 |
|  | 0008624 |
|  | 0019717 |
|  | 0050790 |
|  | 0023034 |
|  | 0005515 |
|  | 0007242 |

---

|  |  |
| --- | --- |
| KEGG ID | hsa:5923 |
|  | mmu:19417 |
|  | rno:192213 |

---

|  |  |
| --- | --- |
| MedLine Reference | 12024021 |
|  | 12477932 |
|  | 1379346 |
|  | 14749369 |
|  | 9933566 |
|  | 10349636 |
|  | 10455104 |
|  | 11042159 |
|  | 11076861 |
|  | 11172053 |
|  | 11217851 |
|  | 11640934 |
|  | 11749043 |
|  | 11753386 |
|  | 11909944 |
|  | 12095702 |
|  | 12466851 |
|  | 12496377 |
|  | 12740578 |
|  | 12805218 |
|  | 12925882 |
|  | 1376246 |
|  | 1379731 |
|  | 1396590 |
|  | 14676298 |
|  | 15029245 |
|  | 15513915 |
|  | 15629714 |
|  | 16141072 |
|  | 16141073 |
|  | 16236762 |
|  | 16314537 |
|  | 16325815 |
|  | 16407208 |
|  | 16467520 |
|  | 16513268 |
|  | 16723539 |
|  | 17028431 |
|  | 17030618 |
|  | 17075039 |
|  | 8088844 |
|  | 8246988 |
|  | 8293576 |
|  | 8299730 |
|  | 8782830 |
|  | 8889548 |
|  | 9045698 |
|  | 9384379 |
|  | 9501318 |
|  | 9570946 |
|  | 9602157 |
|  | 9603515 |
|  | 9872389 |
|  | 10220378 |
|  | 10681520 |
|  | 10918068 |
|  | 14702039 |
|  | 16855028 |
|  | 7637786 |
|  | 7684828 |
|  | 16969075 |
|  | 7828890 |
|  | 11500497 |
|  | 16921254 |
|  | 17114649 |
|  | 17135267 |
|  | 17141611 |
|  | 17321057 |

---

|  |  |
| --- | --- |
| Organism | Homo sapiens {Organism urn:agi-taxid:9606} |
|  | Mus musculus {Organism urn:agi-taxid:10090} |
|  | Rattus norvegicus {Organism urn:agi-taxid:10116} |
|  | Homo sapiens |
|  | Mus musculus |
|  | Rattus norvegicus |
|  | Bos taurus |
|  | Danio rerio |

---

|  |  |
| --- | --- |
| Mouse chromosome position | 9 47.31 cM |
|  | 9 50.0 cM |

---

|  |  |
| --- | --- |
| OMIM ID | 606600 |

---

|  |  |
| --- | --- |
| Rat chromosome position | 8q31 |

---

|  |  |
| --- | --- |
| Hugo ID | 9875 |
|  | HGNC:9875 |

---

|  |  |
| --- | --- |
| Human chromosome position | 15q25.1 |
|  | 15q24.2 |
|  | 15q24 |

---

|  |  |
| --- | --- |
| Swiss-Prot Accession | Q8IUU5 |
|  | A8K270 |
|  | Q13972 |
|  | Q13972.2 |
|  | Q9QZR7 |
|  | B2RS27 |
|  | P27671 |
|  | P27671.2 |
|  | P28818 |
|  | Q5BJV1 |
|  | P28818.1 |
|  | F8VPA5 |
|  | H0YKF2 |
|  | J3KQP9 |
|  | Q16027 |
|  | Q71RC4 |
|  | P70317 |
|  | Q3UY04 |
|  | Q9QYS5 |
|  | Q8N849 |
|  | GRP3\_HUMAN |
|  | Q71UW1 |
|  | RGRF1\_MOUSE |
|  | A8K270\_HUMAN |
|  | Q8IUU5\_HUMAN |
|  | B2RS27\_MOUSE |
|  | Q9QZR7\_MOUSE |

---

|  |  |
| --- | --- |
| PIR ID | A38985 |
|  | S28407 |
|  | S29083 |

---

|  |  |
| --- | --- |
| GenBank ID | NC\_000015 |
|  | XM\_017022459 |
|  | XP\_016877948 |
|  | XM\_017022456 |
|  | XP\_016877945 |
|  | XM\_017022455 |
|  | XP\_016877944 |
|  | XM\_011521867 |
|  | XP\_011520169 |
|  | NM\_153815 |
|  | NP\_722522 |
|  | NM\_001145648 |
|  | NP\_001139120 |
|  | NM\_002891 |
|  | NP\_002882 |
|  | XM\_017022458 |
|  | XP\_016877947 |
|  | XM\_017022457 |
|  | XP\_016877946 |
|  | XM\_011521866 |
|  | XP\_011520168 |
|  | AC011944 |
|  | AC104231 |
|  | CH471136 |
|  | EAW99141 |
|  | EAW99142 |
|  | LS482415 |
|  | SPT35792 |
|  | AK092325 |
|  | AK226101 |
|  | AK290135 |
|  | BAF82824 |
|  | AK301205 |
|  | BAH13432 |
|  | AK309047 |
|  | AL109674 |
|  | AL109675 |
|  | AL355696 |
|  | AL359931 |
|  | BC040275 |
|  | AAH40275 |
|  | DA126882 |
|  | L26584 |
|  | AAA58417 |
|  | M91815 |
|  | S62035 |
|  | AAB26881 |
|  | Q13972 |
|  | NC\_000075 |
|  | XR\_001778822 |
|  | XM\_006510875 |
|  | XP\_006510938 |
|  | NM\_011245 |
|  | NP\_035375 |
|  | NM\_001039655 |
|  | NP\_001034744 |
|  | NM\_001357743 |
|  | NM\_001357744 |
|  | AC102545 |
|  | AH008385 |
|  | AAF08011 |
|  | CH466560 |
|  | EDL20898 |
|  | EDL20899 |
|  | U55232 |
|  | AAB18907 |
|  | AF169826 |
|  | AAD55995 |
|  | AK038936 |
|  | AK079638 |
|  | AK135070 |
|  | BAE22409 |
|  | AK140227 |
|  | BAE24289 |
|  | AK140380 |
|  | AK162908 |
|  | BAE37111 |
|  | BC042449 |
|  | BC138682 |
|  | AAI38683 |
|  | BC138683 |
|  | AAI38684 |
|  | CD775453 |
|  | DV043084 |
|  | L20899 |
|  | AAA02741 |
|  | M91816 |
|  | X59868 |
|  | CAA42525 |
|  | P27671 |
|  | NC\_005107 |
|  | NM\_001105753 |
|  | NP\_001099223 |
|  | NM\_001170531 |
|  | NP\_001164002 |
|  | AC\_000076 |
|  | AABR07071024 |
|  | AABR07071025 |
|  | AABR07071026 |
|  | AABR07071027 |
|  | AABR07071028 |
|  | AAHX01056185 |
|  | AAHX01056186 |
|  | AAHX01056187 |
|  | AAHX01056188 |
|  | AAHX01056189 |
|  | AAHX01056190 |
|  | CH473954 |
|  | EDL77567 |
|  | EDL77568 |
|  | AF044907 |
|  | AAC25083 |
|  | AF044908 |
|  | AAC25084 |
|  | BC091317 |
|  | AAH91317 |
|  | BF405809 |
|  | CB583610 |
|  | CB607130 |
|  | CB713048 |
|  | CB714920 |
|  | X67241 |
|  | CAA47666 |
|  | P28818 |
|  | XM\_011521865 |
|  | XP\_011520167 |
|  | XM\_005254578 |
|  | XP\_005254635 |
|  | XM\_011521864 |
|  | XP\_011520166 |
|  | XM\_011521863 |
|  | XP\_011520165 |
|  | XM\_011521868 |
|  | XP\_011520170 |
|  | XM\_011521869 |
|  | XP\_011520171 |
|  | NC\_018926 |
|  | AMYH02030874 |
|  | AMYH02030875 |
|  | AF370414 |
|  | AAQ15250 |
|  | XM\_011242680 |
|  | XP\_011240982 |
|  | AC\_000031 |
|  | AAHY01080404 |
|  | AAHY01080405 |
|  | AAHY01080406 |
|  | AAHY01080407 |
|  | AAHY01080408 |
|  | XM\_006720628 |
|  | XP\_006720691 |
|  | XM\_006720629 |
|  | XP\_006720692 |
|  | XM\_006720627 |
|  | XP\_006720690 |
|  | AC\_000147 |
|  | ABBA01060598 |
|  | ABBA01060599 |
|  | ABBA01060600 |
|  | ABBA01060601 |
|  | ABBA01060602 |
|  | ABBA01060603 |
|  | XR\_379405 |
|  | NT\_010194 |
|  | NW\_925907 |
|  | NW\_001838219 |
|  | AC\_000058 |
|  | Q71RC4 |
|  | Q8IUU5 |
|  | NT\_039476 |
|  | NW\_001030918 |
|  | AF094687 |
|  | AF094688 |
|  | AF094690 |
|  | AF094691 |
|  | P70317 |
|  | Q3UY04 |
|  | Q9QYS5 |
|  | Q9QZR7 |
|  | Q8N849 |
|  | AAH42449 |
|  | Q8CFQ5 |
|  | NW\_000355 |
|  | XM\_236468 |
|  | XP\_236468 |
|  | 121515 |
|  | 3282806 |
|  | 3282808 |
|  | 57665 |
|  | 60688449 |
|  | 81864892 |
|  | 81909466 |
|  | NW\_001084876 |
|  | NW\_047800 |
|  | Q5BJV1 |
|  | Q71UW1 |
|  | XM\_001063727 |
|  | XM\_001063783 |
|  | XP\_001063727 |
|  | XP\_001063783 |
|  | 123792084 |
|  | 1669492 |
|  | 1708003 |
|  | 27503719 |
|  | 388247 |
|  | 50358 |
|  | 5917696 |
|  | 6424497 |
|  | 74148694 |
|  | 74149246 |
|  | 74187954 |
|  | 81861629 |
|  | 81869484 |
|  | 81900278 |
|  | 81907166 |
|  | 119619547 |
|  | 13124259 |
|  | 25955554 |
|  | 386047 |
|  | 433720 |
|  | 74729359 |
|  | 74759584 |
|  | NT\_086829 |
|  | NC\_007305 |
|  | NW\_001495267 |
|  | XM\_001253580 |
|  | XP\_001253581 |
|  | NC\_007124 |
|  | NW\_001510986 |
|  | XM\_692247 |
|  | XP\_697339 |
|  | 119619548 |
|  | 33341758 |
|  | 74712766 |
|  | 148688951 |
|  | 148688952 |
|  | 149018926 |
|  | 149018927 |

---

|  |  |
| --- | --- |
| Swiss-Prot ID | RGRF1\_HUMAN |
|  | RGRF1\_MOUSE |
|  | RGRF1\_RAT |
|  | B2RS27\_MOUSE |
|  | Q8IUU5\_HUMAN |
|  | Q9QZR7\_MOUSE |
|  | A8K270\_HUMAN |
|  | Q5BJV1\_RAT |

---

|  |  |
| --- | --- |
| Cell Localization | Cytoplasm |
|  | Plasma membrane |

---

|  |  |
| --- | --- |
| Ensembl ID | ENSG00000058335 |
|  | ENSP00000452781.2 |
|  | ENST00000558480.6 |
|  | ENSP00000405963.3 |
|  | ENST00000419573.7 |
|  | ENSP00000378228.3 |
|  | ENST00000394745.3 |
|  | ENSMUSG00000032356 |
|  | ENSMUSP00000034909.4 |
|  | ENSMUST00000034909.10 |
|  | ENSMUSP00000034912.4 |
|  | ENSMUST00000034912.5 |
|  | ENSRNOG00000014025 |
|  | ENSRNOP00000070883.1 |
|  | ENSRNOT00000082757.1 |
|  | ENSP00000452781 |
|  | ENST00000558480 |
|  | ENSP00000405963 |
|  | ENST00000419573 |
|  | ENSP00000378228 |
|  | ENST00000394745 |
|  | ENSMUSP00000034909 |
|  | ENSMUST00000034909 |
|  | ENSMUSP00000034912 |
|  | ENSMUST00000034912 |
|  | ENSRNOP00000070883 |
|  | ENSRNOT00000082757 |
|  | ENSRNOP00000018794 |
|  | ENSRNOT00000018794 |

---

|  |  |
| --- | --- |
| MGI ID | MGI:99694 |
|  | 99694 |

---

|  |  |
| --- | --- |
| RGD ID | 620395 |

---

|  |  |
| --- | --- |
| Unigene ID | Hs.459035 |
|  | Mm.44561 |
|  | Rn.135776 |
|  | Rn.230019 |
|  | Rn.230593 |
|  | Hs.591111 |
|  | Dr.117676 |
|  | Rn.204769 |

---

|  |  |
| --- | --- |
| Homologene ID | 74972 |

---

|  |  |
| --- | --- |
| IPI ID | IPI00016784 |
|  | IPI00171312 |
|  | IPI00118835 |
|  | IPI00795882 |
|  | IPI00137899 |
|  | IPI00435928 |
|  | IPI00204771 |
|  | IPI00199276 |
|  | IPI00476106 |

---

|  |  |
| --- | --- |
| Microarray ID | 96313\_at |
|  | 215688\_at |

---

|  |  |
| --- | --- |
| Homo sapiens Chromosome position | 15q24 |

---

|  |  |
| --- | --- |
| Ensembl Transcript ID | ENST00000261752 |
|  | ENST00000394745 |

---

|  |  |
| --- | --- |
| FunctionalClass | Ras guanyl-nucleotide exchange factor |
|  | guanyl-nucleotide exchange factor |
|  | Rho guanyl-nucleotide exchange factor |
|  | Ras GTPase activator |
|  | protein C-terminus binding |

---

|  |  |
| --- | --- |
| Bos taurus Chromosome position | 7 |

---

|  |  |
| --- | --- |
| Mus musculus Chromosome position | 9 50.0 cM |

---

|  |  |
| --- | --- |
| KEGG pathway | MAPK signaling pathway |

---

|  |  |
| --- | --- |
| Rattus norvegicus Chromosome position | 8q31 |

---

|  |  |
| --- | --- |
| Ensembl Gene ID | ENSG00000058335 |

---

|  |  |
| --- | --- |
| ProteinAtlas NormalTissue | Tissue: lung; Cell type: pneumocytes; Level: Weak; Expression type: Staining; Reliability: Uncertain |
|  | Tissue: cerebral cortex; Cell type: glial cells; Level: Negative; Expression type: Staining; Reliability: Uncertain |
|  | Tissue: bone marrow; Cell type: hematopoietic cells; Level: Weak; Expression type: Staining; Reliability: Uncertain |
|  | Tissue: cerebral cortex; Cell type: neuronal cells; Level: Moderate; Expression type: Staining; Reliability: Uncertain |
|  | Tissue: cervix |
|  | uterine; Cell type: glandular cells; Level: Weak; Expression type: Staining; Reliability: Uncertain |
|  | Tissue: oral mucosa; Cell type: squamous epithelial cells; Level: Moderate; Expression type: Staining; Reliability: Uncertain |
|  | Tissue: pancreas; Cell type: islets of Langerhans; Level: Moderate; Expression type: Staining; Reliability: Uncertain |
|  | Tissue: rectum; Cell type: glandular cells; Level: Moderate; Expression type: Staining; Reliability: Uncertain |
|  | Tissue: skin; Cell type: epidermal cells; Level: Weak; Expression type: Staining; Reliability: Uncertain |
|  | Tissue: appendix; Cell type: lymphoid tissue; Level: Weak; Expression type: Staining; Reliability: Uncertain |
|  | Tissue: ovary; Cell type: ovarian stroma cells; Level: Moderate; Expression type: Staining; Reliability: Uncertain |

---
